# Supplementary material for: GM-CSF Protects Macrophages from DNA Damage by Inducing Differentiation
Source: Cells. 2022 Mar 9;11(6):935. doi: 10.3390/cells11060935 (PMC8946476; doi:10.3390/cells11060935)
Supplement: Supplementary file 1 [file cells-11-00935-s001.zip › cells-1584534-supplementary.pdf]

Supplementary materials

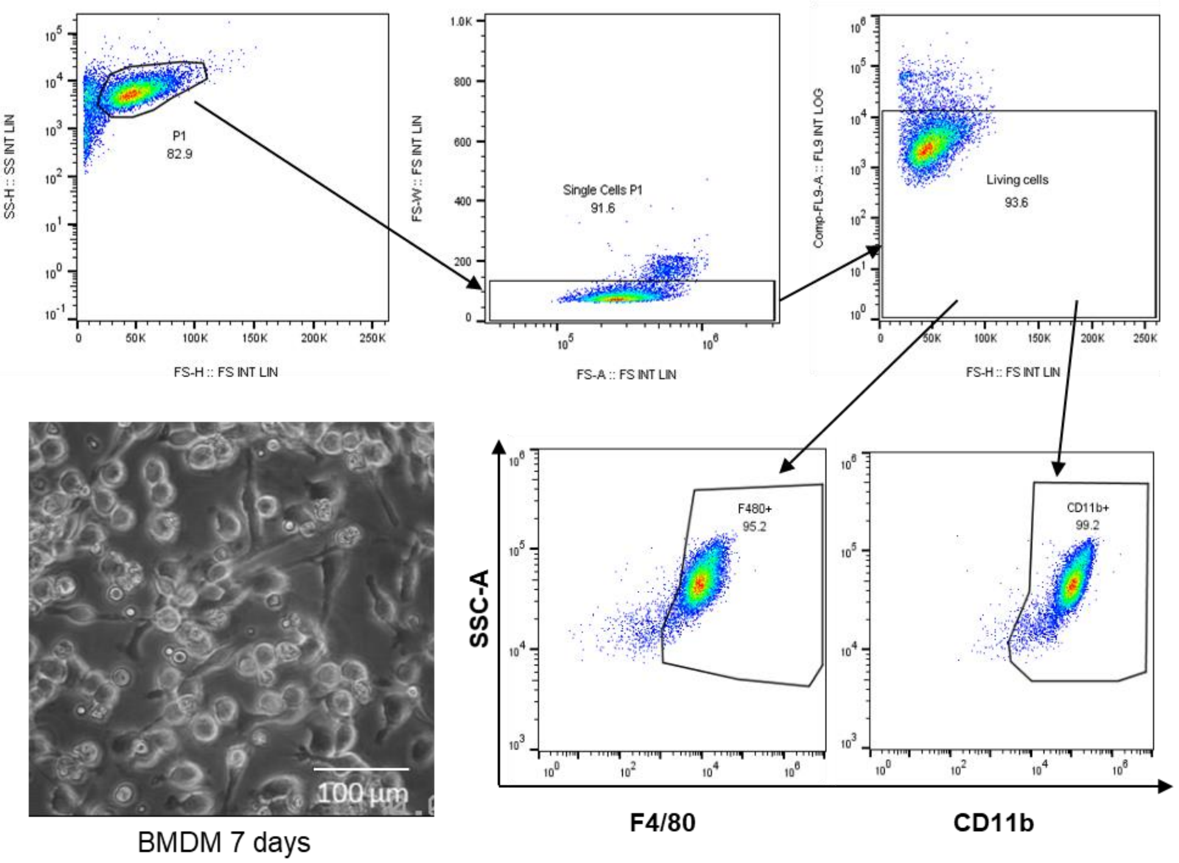

Supplementary Material Figure S1. Gating strategy for BMDM analysis. After forward and side scatter, singlets were selected and gated out dead cells/debris. Then, F4/80 and CD11b was determined.

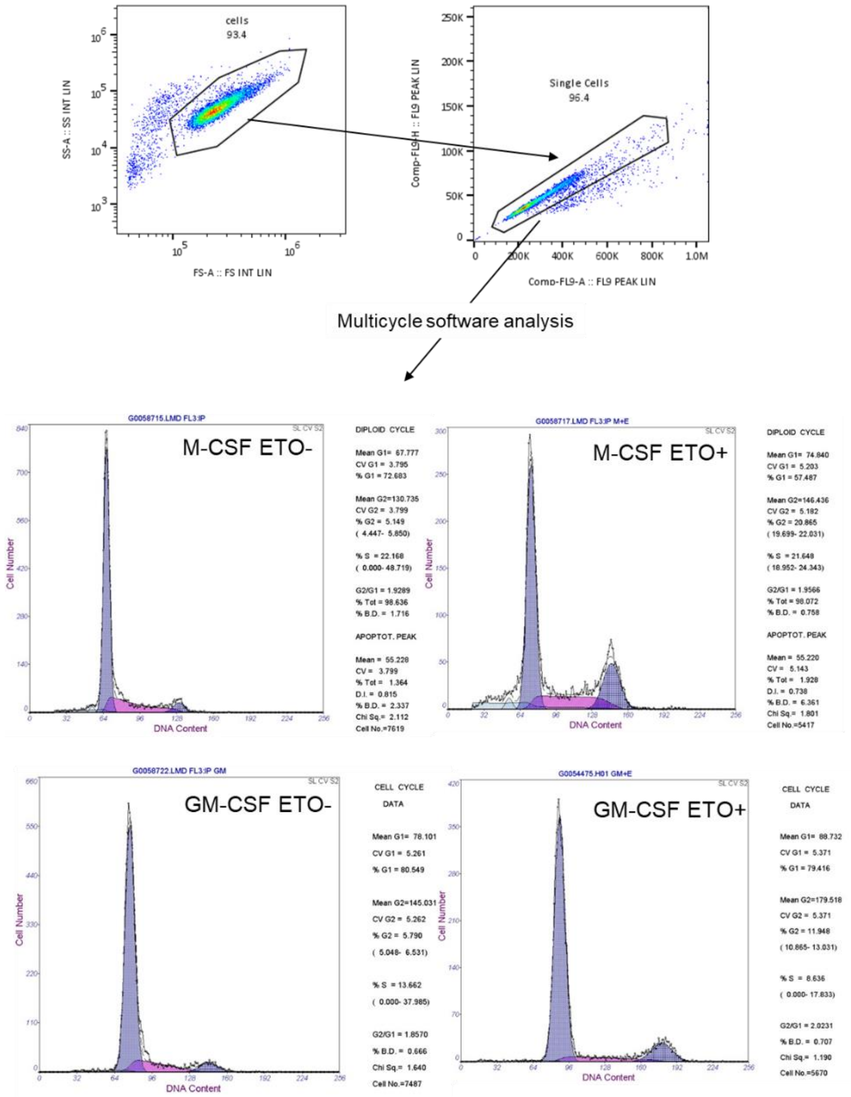

Supplementary Material Figure S2. Gating strategy for cell cycle analysis. After forward and side scatter, singlets were selected. Fluorescence was measured with an Epics Elite flow cytometer (Coulter). 12000 cells were counted for each histogram, and the cell cycle distributions were analyzed with the Multicycle program (Phoenix Flow Systems). The figure includes the histograms of macrophages incubated with M-CSF or GM-CSF, with or without etoposide treatment.

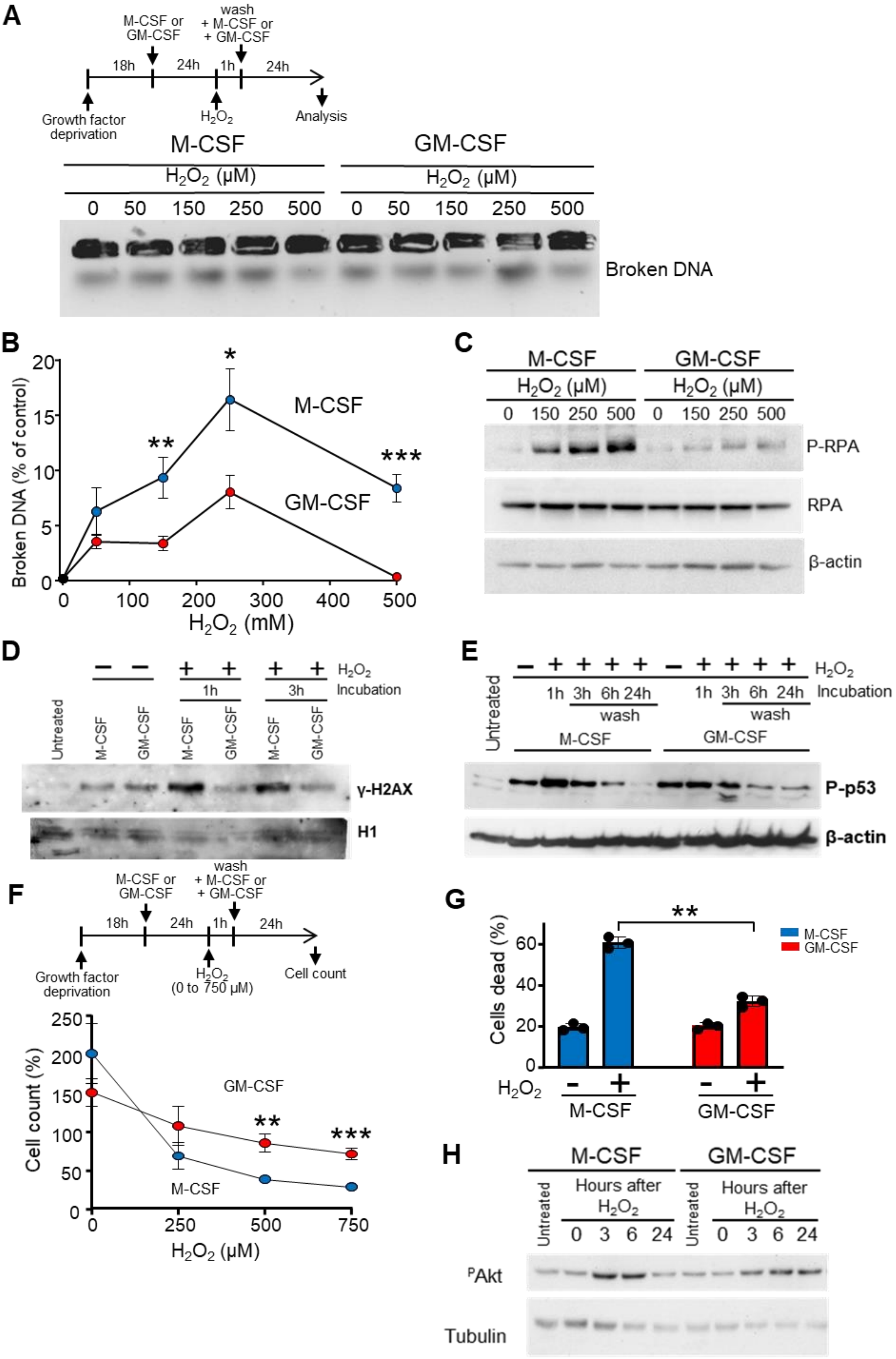

Supplementary Material Figure S3. Hydrogen peroxide induced more double-strand breaks (DSBs) in macrophages incubated with M-CSF in relation to GM-CSF. A) The experimental

design is shown at the top. Macrophages were obtained after 7 d of culture in the presence of M-CSF. Cells were deprived of M-CSF for 18 h and then incubated for 24 h with M-CSF or GM-CSF (10 ng/ml). After that, hydrogen peroxide was added for 1 h at the indicated concentrations, cells were washed and M-CSF or GM-CSF added again for 24 h before analysis. Representative results for DSBs determined using the FAR assay. B) Quantification by densitometry of A (independent experiments, n = 3). C) The experimental design is as Figure 1C, but cells instead of been treated with etoposide were treated with hydrogen peroxide (250 µM). D) The experimental design is as Figure 2A, but cells instead of been treated with etoposide were treated with hydrogen peroxide (250 µM). E) The experimental design is as Figure 2E, but cells instead of been treated with etoposide were treated with hydrogen peroxide (250 µM). F) GM-CSF protects against the effect of hydrogen peroxide on macrophage proliferation. The experimental design is shown at the top. Macrophages were grown in M-CSF or GM-CSF (10 ng/ml) for 24 h, then treated for 1 h with the indicated amounts of hydrogen peroxide, washed and incubated again with M-CSF or GM-CSF for 24 h, and cell counting was performed using a hemocytometer (n=3). G) GM-CSF protects against the effects of hydrogen peroxide on macrophage death. Macrophages were incubated in M-CSF or GM-CSF for 24 h, then treated for 1 h with hydrogen peroxide (250 µM), washed and incubated again with M-CSF or GM-CSF for 24 h, and dead cells (necrotic and apoptotic) were quantified by FACS analysis (n=3). H) The experimental design is as Figure 4B, but cells instead of been treated with etoposide were treated with hydrogen peroxide (250 µM). When indicated the experiments were performed in triplicate, and the results are shown as the mean ± SD. *$p < 0.05$, **$p < 0.01$ and ***$p < 0.001$ in relation to the corresponding treatments with M-CSF or GM-CSF, when all the independent experiments had been compared. Data were analyzed using ANOVA test.

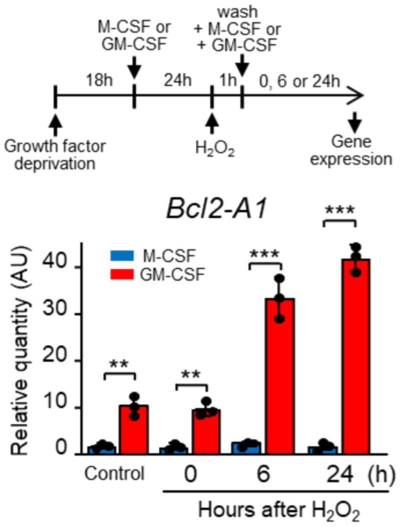

Supplementary Figure S4. The experimental design is shown at the top. Macrophages were incubated in M-CSF or GM-CSF for 24 h, then treated for 1 h with hydrogen peroxide (250 µM), washed and incubated again with M-CSF or GM-CSF for 6 or 24 h, and *Bcl2-A1* gene expression determined (n=3). The experiments were performed in triplicate, and the results are shown as the mean ± SD. **p < 0.01 and ***p < 0.001 in relation to the corresponding treatments with M-CSF or GM-CSF, when all the independent experiments had been compared. Data were analyzed using ANOVA test.

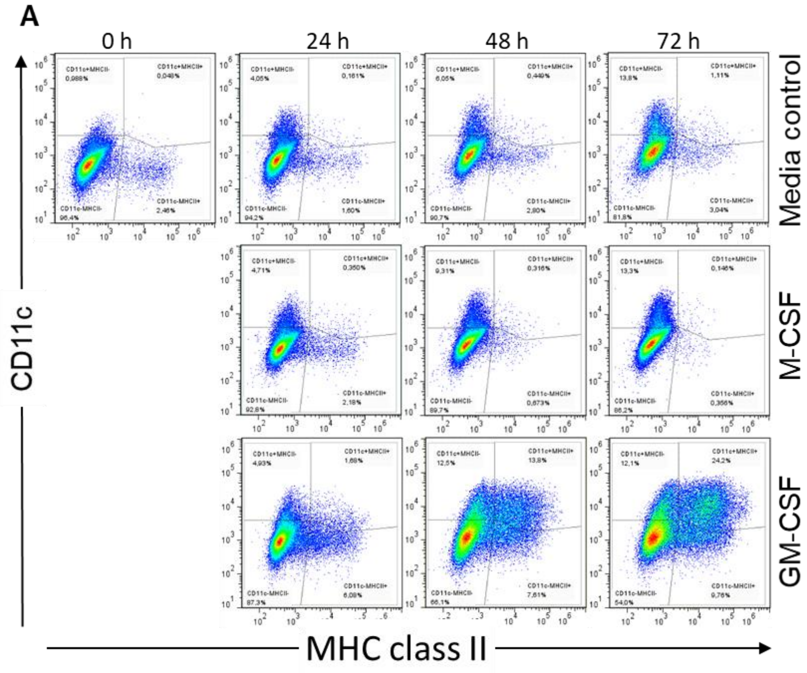

Supplementary Figure S5. Different CD11c and MHCII phenotypes induced in macrophages by M-CSF or GM-CSF. A) Example of characterization of CD11c and MHC II expression.

Macrophages were cultivated in the presence of the indicated growth factors (10 ng/ml) for the indicated times and the surface expression was determined by FACS analysis.

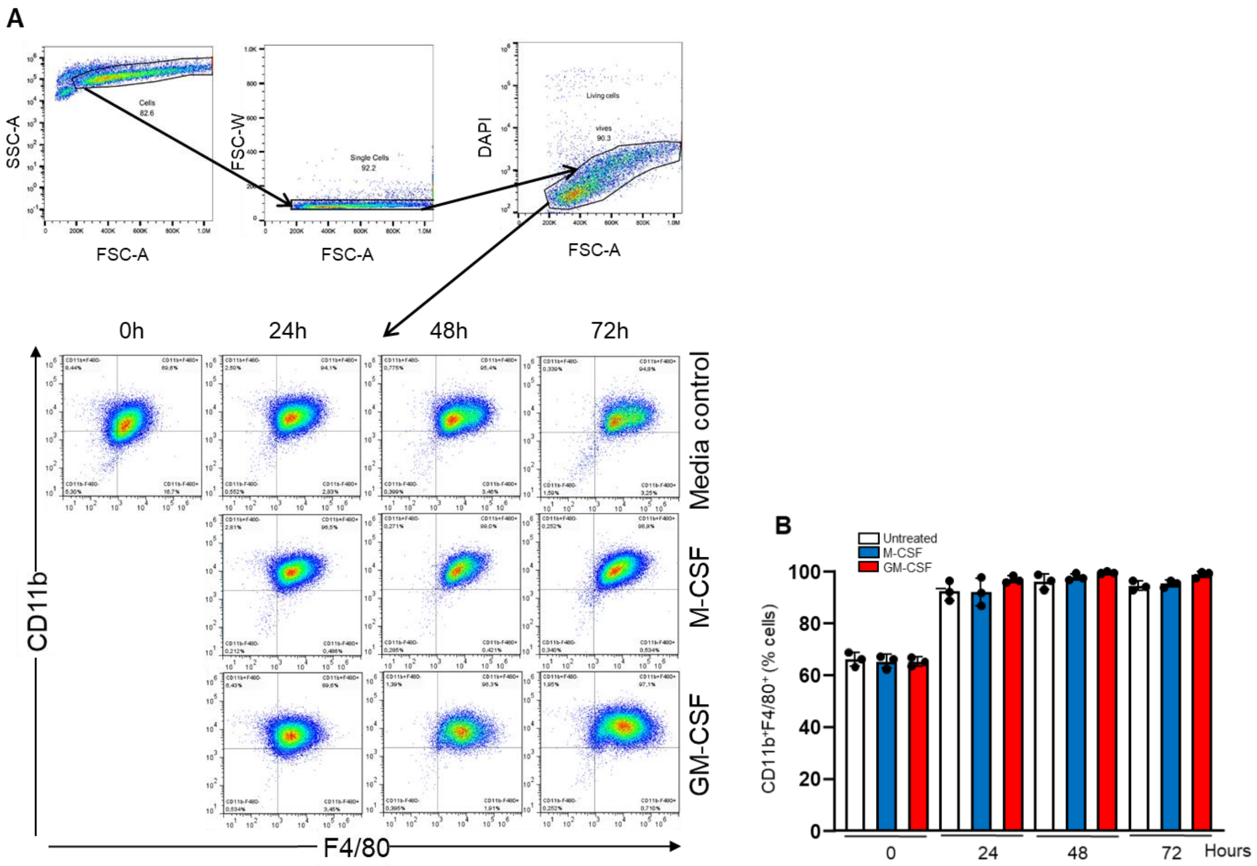

Supplementary Figure S6. Similar CD11b and F4/80 phenotypes induced in macrophages by M-CSF or GM-CSF. A) Gating strategy is shown. After forward and side scatter, singlets were selected. Death cells were excludes using DAPI staining. Fluorescence was measured with an Epics Elite flow cytometer (Coulter). Example of characterization of CD11b and F4/80. Macrophages were cultivated in the presence of the indicated growth factors (10 ng/ml) for the indicated times and the surface expression was determined by FACS analysis. B) Cells expressing double markers (CD11b or F4/80).

Each experiment was performed in triplicate, and the results are shown as the mean ± SD. No significant differences were found in relation to the corresponding treatments with M-CSF or GM-CSF, when all the independent experiments had been compared. Data were analyzed using Student´s t-test.

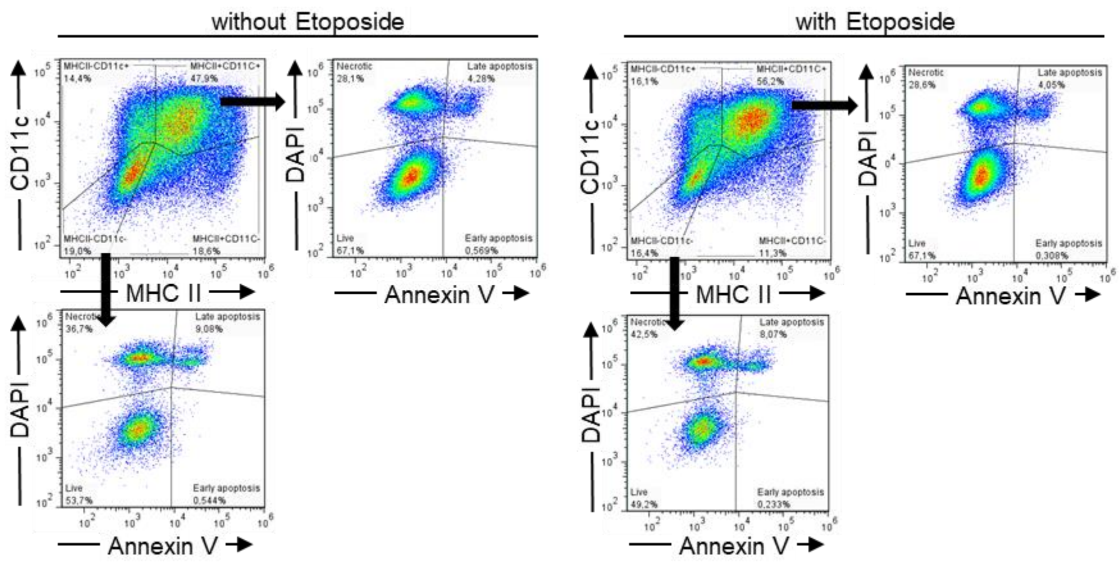

Supplementary Figure S7. Etoposide did not induce apoptosis or necrosis in CD11c- and MHCII-positive macrophages. Example of characterization of apoptosis and necrosis in CD11c- and MHCII-positive macrophages. Macrophages were incubated for 72 h with GM-CSF (10 ng/ml) and then treated or not with etoposide 50 µM for 1 h, washed, and incubated in media containing GM-CSF for 3 h. Double positive cells (CD11c and MHCII) were selected and apoptosis was determined. The numbers of necrotic, apoptotic (early and late) and live cells were calculated using Annexin V and DAPI.

Supplemental Table S1. Primers used for real-time RT-PCR of mRNA.

gene	Forward primer	Reverse primer
Il-1β	5´-TGGGCCTCAAAGGAAAGAAT3'	5'-CAGGCTTGTGCTCTGCTTGT3'
Tnf-α	5´-CCAGACCCTCACACTCAGATC3'	5'-CACTTGGTGGTTTGCTACGAC3'
*P21^waf-1^*	5´-CTGTGGGGTGAGGAGGAGCATGA	5´-ATCACGGCGCAACTGCTCACT
Gadd45	5´-CGAGAAAAGAGAAATCCAAGGC	5´-ATCACGGCGCAACTGCTCACT
Cyclin B1	5´-TTGCTGCTTCTCAAATTGCAGC	5´-CGTTGTCAAGAATTTTCAGCGC
Bcl-2	5´-CGATGGTGTGGTTGCCTTATG	5´-GTCTACTTCCTCCGCAATGCTG
Bcl-X_L	5´-CTGGTCGCCGGAGATAGATT	5´-CCCGGTTGCTCTGAGACATT
Bax	5´-TGAAGACAGGGGCCTTTTTG	5´-AATTCGCCGGAGACACTCG
Bcl-2A1	5´-AAGAGCAGATTGCCCTGGATGT	5´-CCAACCTCCATTCTGCCGTAT
l14	5´-TCCCAGGCTGTTAACGCGGT	5´-GCGCTGGCTGAATGCTCTG
Hprt1,	5´-ATCATTATGCCGAGGATTTGG	5´-GCAAAGAACTTATAGCCCCC
Sdha	5´-TGGGGAGTGCCGTGGTGTCA	5´-CATGGCTGTGCCGTCCCCTG
Mrc1	5´-CGCCCACCAGAGCCCACAAC	5´-TGCTCGCCAGCTCTCCACCT
Msr1	5´-GTGCTGTCTTCTTTACCAGCA	5´-ATGCTGTCATTGAACGTGCG

Bcl-2 (B cell leukemia/lymphoma 2), Bcl-x_L (BCL2-like 1), Bax (Bcl-2-associated X protein), Bcl-2A1 (Bcl2a1, B cell leukemia/lymphoma 2 related protein A1), and L14 (60S ribosomal protein L14).

Supplementary Material Table S2. Antibodies: identification, source, application and dilution used.

Antibody	Identifier	Source	Application	Dilution
Mouse anti-γ-H2AX IgG1 (Ser139)	Cat# 05-636 RRID: AB_309864	UpState	Western blot	1:1000
Mouse anti-histone H1 IgG2a κ	Cat# sc-8030 RRID: AB_675641	Santa Cruz	Western blot	1:1000
Rabbit anti-phospho-Akt (Ser 473) IgG	Cat# 9271 RRID: AB_329825	Cell Signaling	Western blot	1:1000
Mouse anti-α tubulin IgG	Cat# B-5-1-2 T5168; RRID: AB_477579	Sigma-Aldrich	Western blot	1:1000
Mouse anti-p-p53 (Ser15) IgG1	Cat# 9286 RRID: AB_331741	Cell Signaling	Western blot	1:1000
Rabbit anti-RPA IgG	Cat# sc-25376 RRID: AB_655800	Santa Cruz	Western blot	1:1000
Rabbit anti-RPA32/RPA2 (phospho S4, S8) IgG	Cat# A300-245A RRID: AB_210547	Bethyl	Western blot	1:1000
Mouse anti β-actin IgG1	Cat# A5441; RRID: AB_476744	Sigma-Aldrich	Western blot	1:1000
Goat anti-mouse HRP-conjugated IgG	Cat# ab6789; RRID: AB_955439	Abcam	Western blot	1:1000
Goat anti-rabbit HRP-conjugated IgG	Cat# 111-035-003;RRID: AB_2313567	Jackson	Western blot	1:1000
Rabbit anti NBS1 IgG	Cat# NB100-143, RRID: AB_10078050	Novus Biologicals	Western blot	1:1000
Rat anti-CD16/32 IgG2b, κ	Cat# 553141; RRID: AB_394656	BD Pharmigen	Cytometry	1:50
Rat anti-F4/80, IgG2a κ-APC	Cat# 17-4801-82; RRID: AB_2784647	ThermoFisher	Cytometry	1:500
Rat anti-CD11b, IgG2b κ	Cat# 553310; RRID: AB_394774	BD Biosciences	Cytometry	1:500
Hamster anti-CD11c IgG1 λ-PE	Cat# 561044 RRID: AB_2033996	BD Biosciences	Cytometry	1:500
Hamster anti-CD11c, IgG-Alexa Fluor 700	Cat# 56-0114-82 RRID: AB_493992	ThermoFisher	Cytometry	1:500
Rat anti-MHCII, IgG2b κ-FITC	Cat# SAB4700663 RRID: AB_10895927	Sigma-Aldrich	Cytometry	1:500
Rat anti-MHC Class II IgG2b, κ-APC	Cat# 17-5321-82 RRID: AB_469455	ThermoFisher	Cytometry	1:500
Rat IgG2b κ-PE	Cat# 553989 RRID: AB_479625	BD Bioscience	Cytometry	1:500
Rat IgG2a κ-APC	Cat# 17-4321-81 RRID: AB_470181	ThermoFisher	Cytometry	1:500
Rat IgG2b κ-APC	Cat# 17-4031-82 RRID: AB_470176	ThermoFisher	Cytometry	1:500
Hamster IgG λ-PE	Cat# 561022 RRID: AB_647251	BD Bioscience	Cytometry	1:500
Hamster IgG Alexa Fluor 700	Cat# 56-4888-80 RRID: AB_494080	ThermoFisher	Cytometry	1:500
